# Inhibition of CDC25B With WG-391D Impedes the Tumorigenesis of Ovarian Cancer

**DOI:** 10.3389/fonc.2019.00236

**Published:** 2019-04-08

**Authors:** Yangjiong Xiao, Yang Yu, Dan Gao, Wangrui Jin, Pengcheng Jiang, Yuhong Li, Chao Wang, Yuning Song, Peng Zhan, Fei Gu, Cancan Zhang, Bin Wang, Yihua Chen, Bing Du, Rong Zhang

**Affiliations:** ^1^Department of Obstetrics and Gynecology, Shanghai Fengxian District Central Hospital of Southern Medical University, Shanghai, China; ^2^Shanghai Key Laboratory of Regulatory Biology, Institute of Biomedical Sciences and School of Life Sciences, East China Normal University, Shanghai, China; ^3^Department of Gynecology, Changzhou Second People's Hospital Affiliated to Nanjing Medical University, Changzhou, China; ^4^Department of Gynecology, The International Peace Maternity & Child Health Hospital, The China Welfare Institute, Shanghai Jiaotong University, Shanghai, China; ^5^Department of Clinical Pharmacy, Qilu Hospital of Shandong University, Jinan, China; ^6^Department of Medicinal Chemistry, Key Laboratory of Chemical Biology (Ministry of Education), School of Pharmaceutical Sciences, Shandong University, Jinan, China

**Keywords:** WG-391D, ovarian cancer, PDX, primary cell lines, target therapy

## Abstract

Novel inhibitors are urgently needed for use as targeted therapies to improve the overall survival (OS) of patients with ovarian cancer. Here, we show that cell division cycle 25B (CDC25B) is over-expressed in ovarian tumors and associated with poor patient prognosis. All previously reported CDC25B inhibitors have been identified by their ability to reversibly inhibit the catalytic dephosphorylation activity of CDC25B *in vitro*; however, none of these compounds have entered clinical trials for ovarian cancer therapy. In this study, we synthesized a novel small molecule compound, WG-391D, that potently down-regulates CDC25B expression without affecting its catalytic dephosphorylation activity. The inhibition of CDC25B by WG-391D is irreversible, and WG-391D should therefore exhibit potent antitumor activity against ovarian cancer. WG-391D induces cell cycle progression arrest at the G2/M phase. Half maximal inhibitory concentration (IC_50_) values of WG-391D for inhibition of the proliferation and migration of eight representative ovarian cancer cell lines (SKOV3, ES2, OVCAR8, OVTOKO, A2780, IGROV1, HO8910PM, and MCAS) and five primary ovarian tumor cell lines (GFY004, GFY005, CZ001, CZ006, and CZ008) were lower than 10 and 1 μM, respectively. WG-391D inhibited tumor growth in nude mice inoculated with SKOV3 cells or a patient-derived xenograft (PDX). The underlying mechanisms were associated with the down-regulation of CDC25B and subsequent inactivation of cell division cycle 2 (CDC2) and the serine/threonine kinase, AKT. In conclusion, this study demonstrates that WG-391D exhibits strong antitumor activity against ovarian cancer and indicates that the down-regulation of CDC25B by inhibitors could provide a rationale for ovarian cancer therapy.

## Introduction

Ovarian cancer is one of the most common malignances in females. The American Cancer Society estimated that there were 22,240 cases of ovarian cancer, and 14,070 deaths, in the United States in 2018 (1). During its early stages, ovarian cancer can be asymptomatic; hence, ~70% of patients have advanced disease when diagnosed and ultimately develop chemotherapeutic drug resistance and tumor recurrence (2). The death rate from ovarian cancer worldwide has remained as high as 60% over the previous two decades, giving this cancer the highest mortality rate of all gynecological malignances (1, 3). Novel reagents or treatment strategies, particularly for tumors resistant to chemotherapy, are therefore needed to improve the outcomes of surgery and chemotherapy in ovarian cancer patients (2).

As an increasing number of mechanisms underlying ovarian carcinogenesis and progression are being uncovered, opportunities arise for the development of novel therapeutic drugs with which to improve patient survival by targeting specific cancer-associated molecules. In preclinical and clinical studies, a growing number of drugs and strategies have been adopted to prevent disease progression, inhibit recurrence, and reverse drug resistance to chemotherapeutic agents, such as carboplatin and/or paclitaxel. Most anti-cancer therapies under clinical or preclinical development primarily target molecules which play critical roles in malignant transformation, including cancer cell proliferation, invasion, angiogenesis, and stress responses (4). Olaparib, an enzyme poly ADP ribose polymerase (PARP) inhibitor, was approved by the FDA in 2014, and has been one of the most impressive successes in the development of small molecule compounds for ovarian cancer chemotherapy. PARP is a key factor in the repair of single-strand DNA breaks (5) and the inhibition of PARP causes massive DNA damage, resulting in cell cycle arrest and apoptosis; however, the reported immature overall survival (OS) was not significantly different when compared between Olaparib therapy and placebo groups (58% maturity and 24% maturity in phases 2 and 3, respectively) (6, 7). The folate receptor (FR) plays an important role in transporting folate into cells for use in DNA synthesis, repair and methylation. Anti-FR antibody and a folate-conjugated vinca alkaloid are the two main therapeutic strategies that have been included in clinical trials targeting FR (4). Unfortunately, these two strategies both failed in phase III clinical trials (4, 8). Vascular endothelial growth factor (VEGF), platelet-derived growth factor (PDGF), fibroblast growth factor (FGF), and angiopoietin (Ang 1 and 2) are key factors involved in angiogenesis (9). However, a total of ten anti-angiogenic trials in phase II and III, which enrolled 7,530 patients with gynecological cancers, showed only a slight increase in progression-free survival (PFS) of 3.3 months, relative to control groups (9). PD-L1 is abundantly expressed in cancer and tumor-infiltrating immune cells in the tumor microenvironment and suppresses T cell activation (10). PD-1/PD-L1 antibodies, which are able to block the suppressive signaling cascade, have been tested extensively for the treatment of various cancers, including ovarian cancer. The response rates of ovarian patients to PD-1 or PD-L1 antibodies ranged from 6 to 15%, and the median duration of PFS was only ~6 months (11, 12). In summary, although strenuous efforts have been made to develop chemo- and immuno-therapy strategies, the OS of patients with ovarian cancer has only been extended by only a small margin. Hence, novel strategies against ovarian cancer are urgently needed to improve OS of patients.

In this study, we found that CDC25B was over-expressed in ovarian tumors compared with normal ovarian tissues. Furthermore, according to analysis using the Kaplan Meier-plotter online tool, CDC25B expression was positively associated with poor prognosis in ovarian cancer patients. These data strongly indicate that CDC25B plays a critical role in the occurrence and metastasis of ovarian cancer. Hence, CDC25B is a potentially valuable target in the development of chemotherapeutics for the treatment of ovarian cancer. CDC25 is a serine and threonine dual specificity phosphatase, which has three isoforms, CDC25A, CDC25B, and CDC25C, in mammalian cells. CDC25B is an initiator of mitosis that plays a key role in controlling G2/M cell cycle phase transition (13–16). CDC25B dephosphorylates and activates CDC2, which is required for entry into mitosis, and is over-expressed in various tumors, including breast cancer (17), colorectal carcinoma (18), endometrioid endometrial carcinoma (19), esophageal squamous cell carcinoma (20), pancreatic ductal adenocarcinoma (21), and non-small cell lung carcinoma (22), among others. CDC25B inhibitors, which are reported to inhibit the progression of cancer in a highly efficient manner, are primarily vitamin K analogs (23), NSC 663284 (24), naphthofurandione 3-benzoyl-naphtho[1,2-b]furan-4,5-dione (5169131) (25), adociaquinone B (26), IRC-083864, and BN82685 (27, 28); however, these compounds have mainly been studied in breast cancers, colorectal carcinoma, hematological malignancies, and non-small cell lung carcinoma, while far less is known about the efficiency of these inhibitors in ovarian cancers. All of these compounds were screened out using *in vitro* chemical assays because they reversibly inhibit the catalytic dephosphorylation activity of CDC25B, but none have entered clinical trials for ovarian cancer therapy. Novel CDC25B inhibitors with high efficiency could be very valuable for the clinical treatment of ovarian cancer. Yuning Song and colleagues previously reported a CDC25 inhibitor, CHEQ-2, that not only inhibited the catalytic dephosphorylation activity of CDC25A and CDC25B *in vitro*, but also down-regulated CDC25A and CDC25B levels in cancer cells (29). It would be particularly useful if such inhibitors could avoid the reversion of dephosphorylation activity inhibition because of increased CDC25 protein expression levels.

We hypothesized that CDC25B down-regulators could be valuable for targeted therapies against ovarian cancer. In this study, a novel small molecule compound, WG-391D, synthesized in our laboratory, was found to efficiently inhibit the expression, but not the catalytic dephosphorylation activity, of CDC25B in ovarian cancer cells. Hence, WG-391D could represent a potent inhibitor of ovarian cancer tumorigenesis. Our data show that WG-391D not only inhibited ovarian cancer cell proliferation and migration *in vitro*, but also impeded tumor growth in BALB/c athymic nude mice inoculated with ovarian cancer cells or patient-derived xenograft (PDX) tissues. The mechanism underlying these activities was associated with the down-regulation of CDC25B and the inactivation of CDC2 and AKT. Overall, this study demonstrates that WG-391D can efficiently inhibit ovarian cancer tumorigenesis by down-regulating CDC25B. WG-391D therefore has potential as an ideal lead compound for the development of chemotherapeutics against ovarian cancer. This study is the first to demonstrate a rationale for treating ovarian cancer using CDC25B down-regulators.

## Materials and Methods

### Synthesis of WG-391D

To synthesize WG-391D, the key intermediate, 2-(2-phenylacetyl)-2,3,4,9-tetrahydro-1H-pyrido[3,4-b]indole-8- carboxylic acid, was prepared according to our previous report (30). This was dissolved in anhydrous *N,N*-dimethylformamide, 1-(3-dimethylaminopropyl)-3-ethylcarbodiimide hydro-chloride (EDC·HCl) and *N*-hydroxybenzotriazole (HOBt) was added at 0°C. After stirring for 15 min, 3-amino- propanol was added to the mixture, which was then stirred for a further 3 h, diluted with H_2_O, and extracted using EtOAc. The combined organic phase was then dried over anhydrous Na_2_SO_4_, concentrated, and subjected to chromatography over silica gel, to produce WG-391D as a white powder. Finally, WG-391D, 8-(3-hydroxypropylcarbamoyl)-N-phenylacetyl-1,3,4,9-tetrahydro-beta-carboline, was dissolved in DMSO at a concentration of 50 mM.

### *In vitro* Enzyme Assay

The enzymic inhibition activity of WG-391D was measured using a “Human Protein Phosphatase Cdc25 Combo Fluorometric Assay Kit” (Abnova, Taiwan, China), according to the manufacturer's instructions. In brief, 5 μL of WG-391D (100 μM in DMSO) was added into 40 μL of “Assay Mixture” (30 μL distilled water, 5 μL 10 × assay buffer, and 5 μL 10 × 3-O-methylfluorescein phosphate). The same volume of DMSO and NSC663284 (100 μM in DMSO, Sigma, USA) served as negative and positive controls, respectively. Enzyme reactions were initiated by adding 5 μL of recombinant CDC25B protein and then preincubated at room temperature for 5–8 min. Fluorescence intensity was then measured for 60 min at 5 min intervals, using excitation and emission wavelengths of 485 and 525 nm, respectively.

### Ovarian Cancer and Primary Ovarian Tumor Cell Lines

The human ovarian cancer cell lines, A2780, IGROV-1, SKOV3, MCAS, HO8910PM, ES2, OVTOKO, and OVCAR8, and the ovarian epithelial cell line, HOSEPIC, were cultured in RPMI 1640 (GIBCO, NE, USA), containing 10% fetal bovine serum (FBS) (GIBCO), 100 U/ml penicillin (GIBCO) and 100 μg/ml streptomycin (GIBCO).

In order to prepare primary ovarian cancer cells, tumors that were freshly-derived from patients (identification numbers: GFY005, CZ001, CZ006, and CZ008) undergoing surgery, or from third generation PDX (from patient GFY004) inoculated into BALB/c nude mice, were cut into 1–2 mm diameter pieces and digested using a Tumor Dissociation Kit (130-095-929, Miltenyi, Teterow, Germany) in a water bath at 37°C for 45 min. Single cell suspensions were centrifuged at 150 x g for 5 min, then washed twice with RPMI 1640. Primary ovarian tumor cells were then cultured in RPMI 1640 containing 20% FBS, 100 U/ml penicillin, and 100 μg/ml streptomycin. All primary tumor cell lines were cultured for 5 to 10 generations prior to experiments. Specific patient characteristics are detailed in Table 1.

**Table 1:** Characteristics of participants with ovarian cancer.

	**CZ001**	**CZ006**	**CZ008**	**GFY004**	**GFY005**
Tumor type	Clear cell carcinoma	High grade serous carcinoma	Poorly differentiated carcinoma	High grade serous carcinoma	High grade serous carcinoma
Tumor grade	IC	IIIC		IIIB	IIIC
Tumor derived site	Right ovary	Left fallopian tube	Right ovary	Right ovary	Omental metastasis
Age (years)	48	60	63	60	76
Nationality	Chinese Han	Chinese Han	Chinese Han	Chinese Han	Chinese Han
Sex	Female	Female	Female	Female	Female

### Cell Proliferation Assay

Cells (2 × 10^4^/ml in a total volume of 100 μl per well) were seeded into 96-well plates and cultured overnight. WG-391D was added at a series of concentrations up to 33.33 μM, and cells cultured for a further 72 h. Cell viability was determined by 3- (4,5-dimethylthiazol-2-yl)- 5- (3-carboxymethoxyphenyl)- 2- (4- sulfophenyl)- 2H-tetrazolium (MTS) assay, using a Cell Proliferation Assay kit (#G5430, Promega, Wisconsin, USA), in accordance with the manufacturer's instructions, and IC_50_ values calculated.

### Cell Migration Assay

Transwells, containing polycarbonate membrane filter inserts with 8-μm pore size (Costar Group, DC, USA), were inserted into 24 well cell culture plates. Cells (5 × 10^4^ in 200 μl RPMI 1640, without FBS, per well) were seeded into the upper chambers. RPMI 1640 containing 10 % FBS (700 μl per well) was added into the 24 well plates. Cells were cultured for 18 h. After fixing with 4 % paraformaldehyde, cells were stained with 2% crystal violet. Non-migrating cells on the upper chamber surface were scraped out and five random fields of migrated cells on the underside of the membrane were photographed under a microscope and migrated cells were counted.

### Cell Cycle Assay

SKOV3 cells (1 × 10^5^/ml in 2 ml per well) were seeded into 6-well plates and cultured overnight. WG-391D was added at the indicated concentrations. Cells were cultured for a further 6 to 48 h. After fixing in 70% ethanol at 4°C for 24 h, cells were stained with 50 μg/ml propidium iodide (PI), containing 400 U/ml RNase A, for 30 min at room temperature in the dark. Cells were measured using a FACSCalibur flow cytometer (BD Biosciences, CA, USA) within 2 h of staining. Cell cycle distribution was analyzed using ModFit LT software (Verity Software House, ME, USA).

### Cell Apoptosis Assay

SKOV3 cells (1 × 10^5^/ml in a 2 ml per well) were seeded into 6-well plates and cultured overnight. WG-391D was added at increasing concentrations, from 0.2 to 25 μM. Cells were cultured for a further 6 to 24 h and stained using an Annexin V and PI staining kit (V13242, Thermo Fisher Scientific, MA, USA), according to manufacturer's instructions. Apoptotic cells were detected using a FACSCalibur (BD Biosciences) instrument, and analyzed using FlowJo software (FlowJo LLC, OR, USA).

### Xenografts in Nude Mice

In order to generate PDX from patient GFY004, fresh ovarian tumor tissues (derived from surgery) were cut into 1–2 mm samples and punched into the left front flank of BALB/c null nude mice by puncture needles. This represented the first generation of PDX. The diameters of the tumors that develop subsequently, would be 5–10 mm in size 1 month later. Mice were sacrificed 1 month after flank puncture and surgery was carried out to remove tumors. Tumors were cut into 1–2 mm samples and punched into the left front flank of nude mice, as described above; this represented the second generation of PDX models. The same procedure was used to generate a third generation of PDX models.

Five- to six-week-old BALB/c athymic nude mice were inoculated with 1 × 10^6^ SKOV3 cells, or 1–2 mm diameter third generation ovarian PDX from GFY004, under the left front flank. Seven days later, mice were treated with 25 mg/kg/day WG-391D continuously for a further 3 weeks. Tumor volume, and the body weights of the mice, were monitored at intervals of 1 or 2 days. Tumor volume was calculated using the following formula: volume = a × b × b/2, where “a” was the longer diameter and “b” the shorter one. After the mice were sacrificed, tumors were surgically removed and weighed.

### RNA Sequencing

SKOV3 cells were treated with or without 5 μM WG-391D for 24 h. Assays were conducted using triplicate samples. Total RNA was extracted using TRIzol (Thermo Fisher SCIENTIFIC, MA, USA) and RNA sequencing libraries prepared using an Illumina Standard library preparation kit, according to the manufacturer's instructions. mRNA expression levels were determined by RNA sequencing at Shanghai OE Biotech Co., Ltd (Shanghai, China) using the Illumina HiSeq™ 2500 platform, as previously described (31). Gene expression values were normalized as fragments per kilobase of transcript per million mapped reads (FPKM), and *P* < 0.05, generated using the DESeq Software Package (bioconductor.org/), were considered to be statistically significant.

### qRT-PCR

SKOV3 cells were lysed in TRIzol for total RNA extraction after treatment with or without 5 μM WG-391D for 24 h. In order to measure CDC25B mRNA expression levels in various cell lines, SKOV3, OVCAR8, ES2, OVTOKO, A2780, HO8910PM, MCAS, HOSEPIC, IGROV1, GFY004, CZ001, CZ006, and CZ008 cells were lysed in TRIzol without treatment. cDNA was generated using a Superscript III Reverse transcriptase kit (Life Technologies). The expression levels of target genes were then determined by SYBR® Green Real-time PCR master mix kit (Takara, Shiga, Japan) on a 7900HT machine (Applied Biosystems, Foster City, CA, USA). The primer pair, 5′-GCATGGAGAGTCTCATTAGTGC-3′ and 5′-CTCCGCCTCCGCTTATTCT-3′, was used to amplify *CDC25B*. The primers, 5′-ACCCAGAAGACTGTGGATGG-3′ and 5′-TTCAGCTCAGGGATGACCTT-3′ were used to amplify the housekeeping gene *GAPDH* as a control. Relative mRNA expression levels were expressed as the ratio of the levels of target genes to those of *GAPDH* using the ΔΔCt method.

### Western Blotting

Cells or tissues were lysed using RIPA buffer (Beyotime, Jiangsu, China) containing protease and phosphatase inhibitors. Protein concentrations were then measured using a BCA protein assay kit (Thermo Fisher Scientific). A total of 20 μg protein from SKOV3, HO8910PM, CZ001, CZ006, GFY004, and GFY005 cells, and 100 μg total proteins from ovarian tumor tissues, normal ovarian tissues, or xenograft tumors from nude mouse models, were subjected to SDS-PAGE on 10% gels and blotted onto nitrocellulose filter (NC) membrane by electrophoresis.

The following primary antibodies were used: anti-CDC25B (#D260980-0025; Sangon Biotech, Sangon, Shanghai, China); anti-AKT (#8272), anti-pAKT (ser473) (#8271), anti-PARP and –cleaved PARP (#9532), anti-CDC2 (#9116), and anti-pCDC2 (Tyr15) (#4539) (Cell Signaling Technology, Danvers, MA, USA); and anti-GAPDH (#AP0063) (Bioworld Technology, MN, USA). Goat anti-mouse (#926-32210, LI-COR Biosciences, NE, USA) or goat anti-rabbit (#926-32211, LI-COR) IRDye 800CW-labeled secondary antibodies were used for staining and detected using Image Studio Version 5.2 on an Odyssey CLx infrared imaging system (LI-COR).

### Online Database Analysis

Correlations of *CDC25B* mRNA expression levels and PFS or post-progression survival (PPS) of ovarian cancer patients were analyzed using the Kaplan Meier-plotter online database (http://kmplot.com/analysis/index.php?p=service&cancer=ovar). Parameter cut-off values were selected using the auto select best cutoff function. This online tool has been described in a previous study by its creators (32).

Normalized *CDC25B* expression data from 902 ovarian tumors and 51 normal tissues were downloaded from the Gene Expression across Normal and Tumor tissue (GENT) online database (http://medical-genome.kribb.re.kr/GENT/index.php). This public database has been described in a previous study by its creators (33).

### Statistical Analysis

Two to three independent experiments, with at least three replicates, were conducted for all assays. Data are presented as mean ± standard deviation (SD). Figures were plotted using GraphPad Prism 5.0 software (GraphPad Software Inc., CA, USA). The statistical significance of differences between experimental groups was evaluated using GraphPad Prism 5.0 software. The two-tailed Student's *t*-test was performed for data which followed a Gaussian distribution, while the non-parametric Mann-Whitney test was used for non-Gaussian distributed data. Correlations between mRNA expression and the IC50s of WG-391D were analyzed using Pearson's method (for data with a Gaussian distribution) or Spearman's method (for data with a non-Gaussian distribution). Two way analysis of variance (ANOVA) was used to statistically analyze CDC25B catalytic dephosphorylation activity. A *P* < 0.05 was considered to be statistically significant.

## Results

### The Expression of CDC25B Is Highly Up-Regulated in Ovarian Cancer

**Figure 1:**
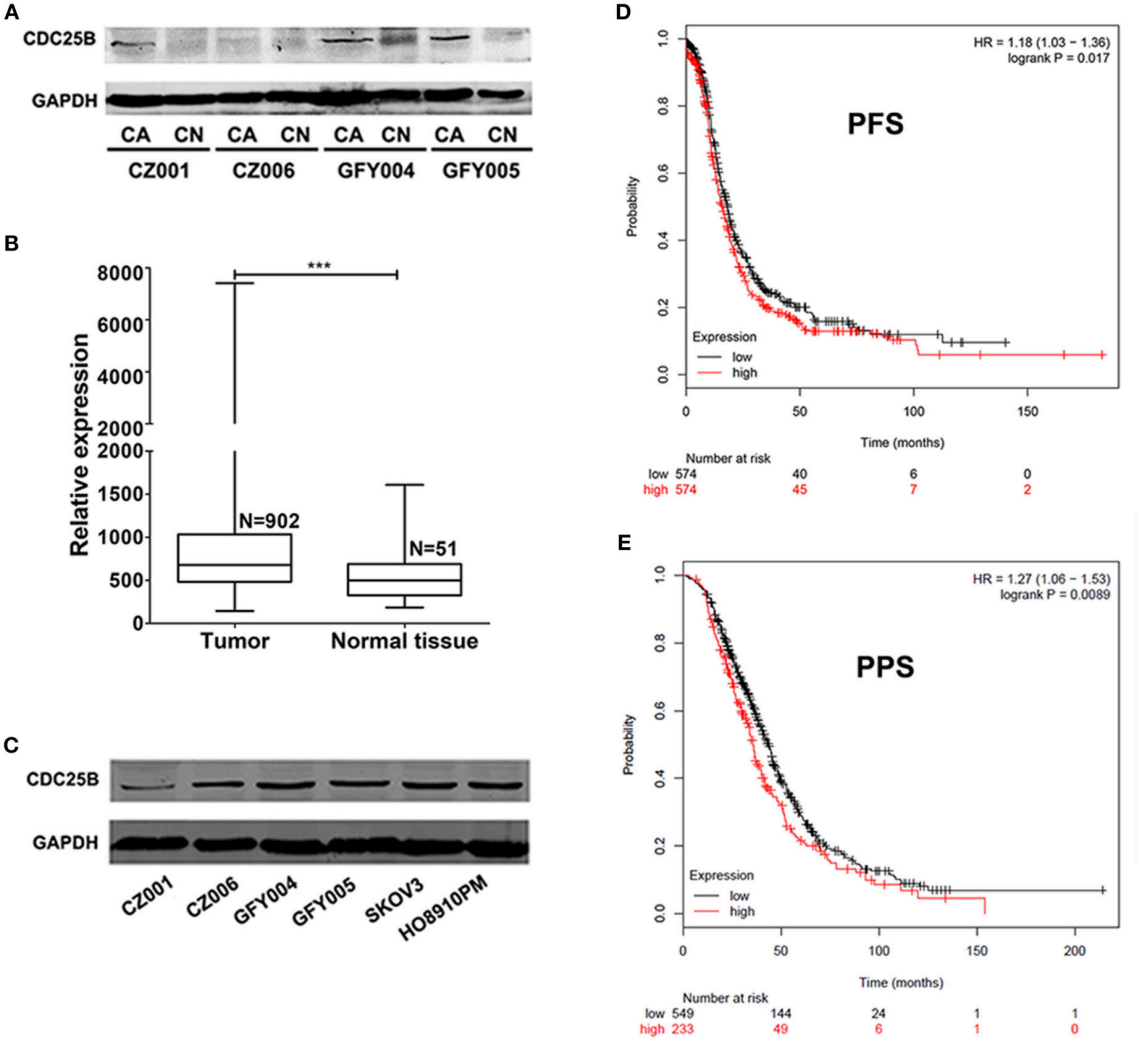
CDC25B is over-expressed in ovarian tumors and ovarian cancer cell lines. **(A)** CDC25B expression was detected in ovarian tumor and normal ovarian tissue samples from the same patients by western blot assay. Total proteins (100 μg per sample) were analyzed. CA, carcinoma; CN, control. **(B)** Statistical analysis of *CDC25B* expression levels in tumor and normal tissue samples using data down-loaded from the GENT database. N, sample number. ***p < 0.001. **(C)** CDC25B expression levels were detected in primary ovarian tumor cell lines (CZ001, CZ006, GFY004, and GFY005) and ovarian cancer cell lines (SKOV3 and HO8910PM) by western blot assay. Total proteins (20 μg per sample) were analyzed. **(D,E)** Analysis of the relationship between CDC25B expression levels and ovarian cancer patient progression free survival periods (PFS) and post progression survival (PPS) periods using the Kaplan Meier-plotter website.

CDC25B expression levels were detected by western blotting in both ovarian tumors and normal ovarian tissue samples from the same patients. As shown in Figure 1A, CDC25B was over-expressed in ovarian tumors compared with normal ovarian tissues. The characteristics of ovarian cancer patients are presented in Table 1. To confirm the over-expression of CDC25B in ovarian tumors, normalized *CDC25B* expression data from 902 ovarian tumor and 51 normal tissue samples were downloaded from the GENT online database (33). As shown in Figure 1B, *CDC25B* was also significantly over-expressed in these ovarian tumor samples compared with normal tissues, *p* < 0.001. Also, high CDC25B expression levels were universally detected in both primary ovarian cancer cell lines (GFY004, GFY005, CZ001, and CZ006) and representative ovarian cancer cell lines (SKOV3 and HO8910PM) (Figure 1C). Analysis of mRNA array data (32), using the Kaplan Meier-plotter online database, showed that CDC25B overexpression was positively associated with poor PFS (Figure 1D) and PPS (Figure 1E) of ovarian cancer patients.

### WG-391D Inhibits the Expression of CDC25B in an Efficient Manner

**Figure 2:**
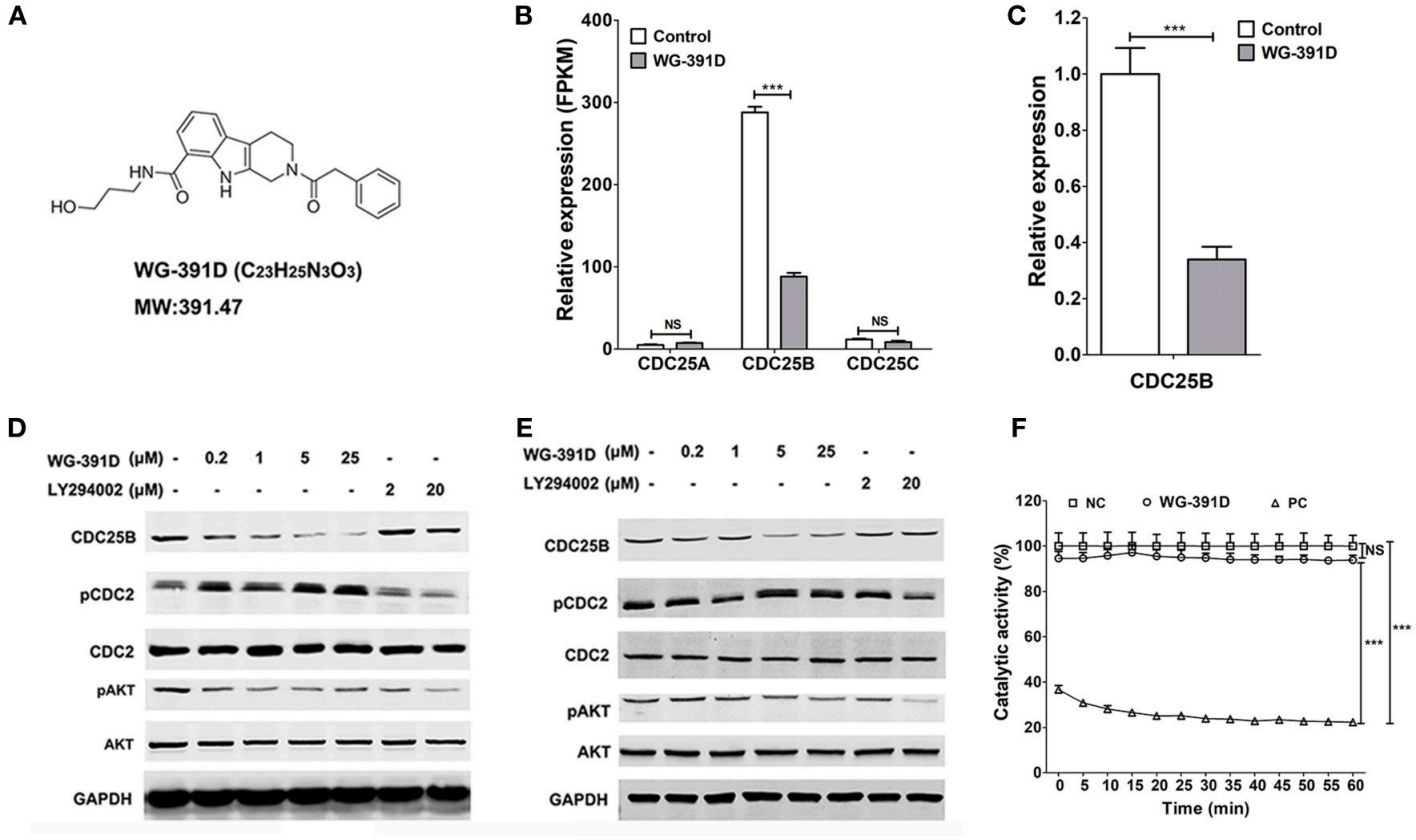
WG-391D down-regulates CDC25B and inactivates CDC2 and AKT. **(A)** Molecular structure of WG-391D. **(B)** RNA sequencing analysis of *CDC25A, CDC25B*, and *CDC25C* mRNA expression in SKOV3 cells treated with 5 μM WG-391D for 24 h. **(C)** qRT-PCR detection of *CDC25B* mRNA expression in SKOV3 cells treated with/without 5 μM WG-391D for 24 h. **(D)** CDC2 and AKT expression and/or activation was evaluated in SKOV3 cells treated with a series of concentrations of inhibitor for 24 h. **(E)** CDC2 and AKT expression and/or activation was evaluated in GFY004 cells treated with a series of concentrations of inhibitor for 24 h. Expression levels of CDC25B, CDC2, AKT, and GAPDH were detected, as indicated. Phosphorylation of CDC2 and AKT were detected (pCDC2 and pAKT, respectively). GAPDH served as a house-keeping control. Total proteins (20 μg per sample) were analyzed by SDS-PAGE. **(F)** Effect of WG-391D (10 μM) on CDC25B dephosphorylation catalytic activity was determined by *in vitro* enzyme assay. NC, DMSO served as the negative control. PC, NSC663284 served as the positive control. NS, not significant. ***p < 0.001.

WG-391D is a novel small molecule compound, which was synthesized in our laboratory. The molecular formula and expected molecular weight of WG-391D are C_23_H_25_N_3_O_3_ and 391.47 Daltons, respectively. The molecular structure of WG-391D is shown in Figure 2A. RNA sequencing data showed that WG-391D significantly inhibited *CDC25B* mRNA expression without affecting that of *CDC25A* and *CDC25C* (Figure 2B). According to KEGG pathway analysis, which was carried out on our RNA sequencing data, CDC25B is the only gene that can be significantly inhibited by WG-391D among the genes involved in the promotion of G2/M phase transition (Figure S1). qRT-PCR assays confirmed that *CDC25B* was down-regulated by WG-391D (Figure 2C). Moreover, WG-391D reduced CDC25B protein levels in a dose-dependent manner (Figures 2D,E) and markedly reduced CDC25B protein levels at concentrations as low as 0.2 μM in SKOV3 cells (Figure 2B). As shown in Figures 2D,E, CDC25B down-regulation caused increased phosphorylation of CDC2, a downstream molecular target of CDC25B. CDC25B is known to be able to activate the serine/threonine kinase AKT in cancer cells treated with the mTOR inhibitor, rapamycin (34). Our data demonstrated that downregulation of CDC25B inhibited AKT phosphorylation. CDC2 phosphorylation and AKT dephosphorylation resulted in inactivation of CDC2 and AKT, respectively (Figures 2D,E). In the present study, we used an *in vitro* chemical assay to detect the effects of WG-391D on CDC25B catalytic dephosphorylation activity. As shown in Figure 2F, WG-391D had no effect upon CDC25B catalytic activity.

### WG-391D Efficiently Inhibits the Proliferation and Migration of Ovarian Cancer Cells

**Figure 3:**
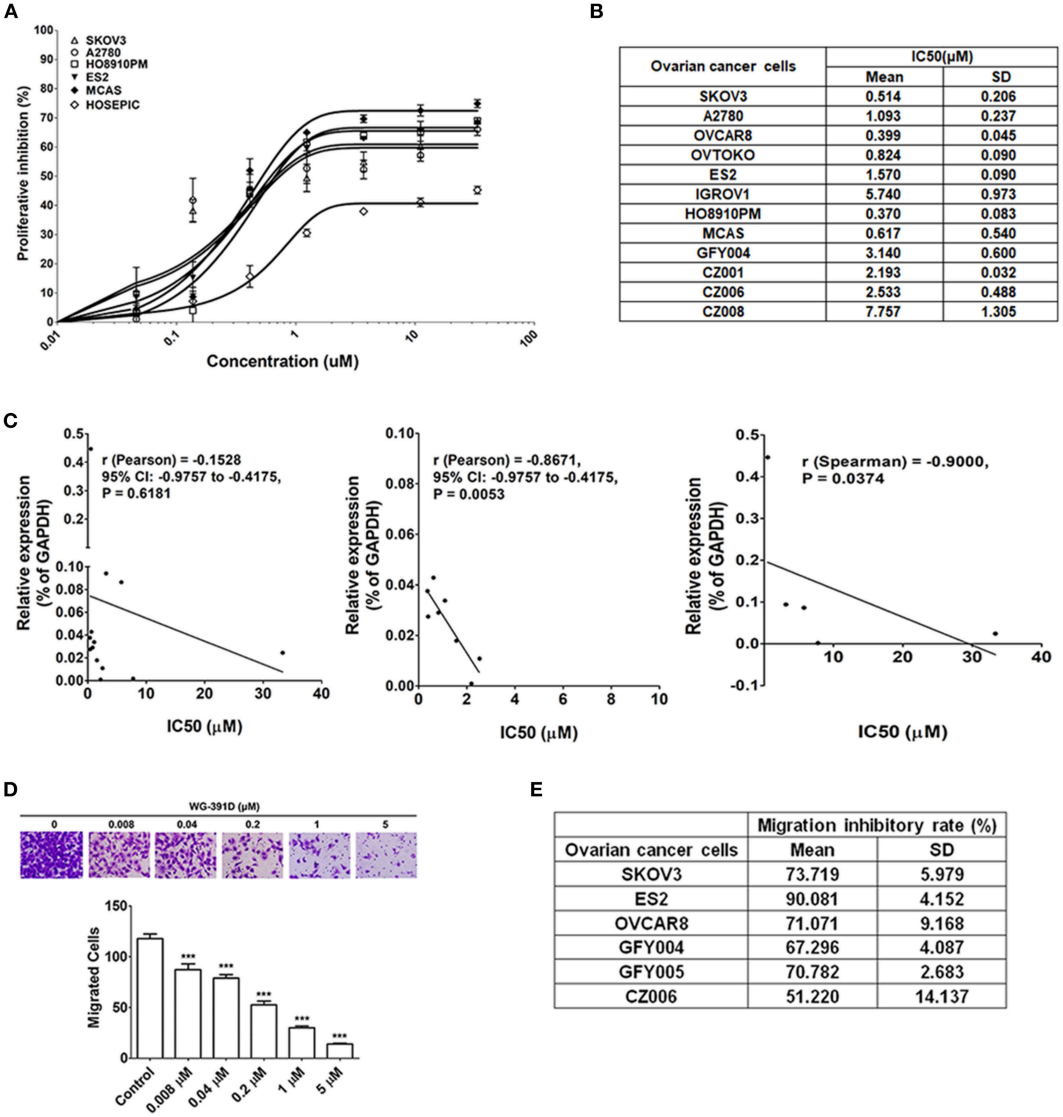
WG-391D strongly inhibits ovarian cancer cell proliferation and migration. **(A)** Cell lines were treated with increasing concentrations of WG-391D and cell viability determined by MTS assay. Error bars, mean ± SD. **(B)** IC_50_ values of WG-391D on eight ovarian cancer (SKOV3, OVCAR8, ES2, OVTOKO, A2780, HO8910PM, and MCAS) and four primary ovarian tumor (GFY004, CZ001, CZ006 and CZ008) cell lines were measured by MTS assay. **(C)** Correlations of CDC25B mRNA expression levels (detected by qRT-PCR) with the IC50s of WG-391D were analyzed. Left panel: Correlations of CDC25B expression levels and the IC50s of WG-391D detected in all ovarian cell lines (SKOV3, OVCAR8, ES2, OVTOKO, A2780, HO8910PM, MCAS, HOSEPIC, IGROV1, GFY004, CZ001, CZ006, and CZ008) involved in panels **(A,B)**. Middle panel: Analysis of the correlations between CDC25B expression levels and the IC50s of WG-391D to nine cell lines (OVCAR8, ES2, OVTOKO, A2780, HO8910PM, MCAS, GFY004, CZ001, and CZ006) with low CDC25B expression levels and low WG-391D IC50s. Right panel: Analysis of the correlations between CDC25B expression levels and the IC50s of WG-391D in four cell lines (SKOV3, IGROV1, HOSEPIC and CZ008) with high CDC25B expression or high IC50s for WG-391D. **(D)** The effects of WG-391D on SKOV3 cell migration were measured. Upper panel, representative data showing migrated SKOV3 cells following treatment with different concentrations of WG-391D for 18 h. Lower panel, statistical analysis of SKOV3 cell migration data. **(E)** Migration inhibitory ratios were calculated for various ovarian cancer (SKOV3, ES2, and OVCAR8) and primary ovarian tumor (GFY004, GFY005, and CZ006) cell lines treated with 1 μM WG-391D for 18 h. Untreated cells were used as negative controls. ***p < 0.001.

The effect of WG-391D on ovarian cancer cell proliferation was detected by MTS assays. As shown in Figure 3A, the IC_50_ values of WG-391D for all five representative epithelial ovarian cancer cell lines (SKOV3, A2780, MCAS, ES2, and HO8910PM) were <1.00 μM. Of these ovarian cancer cell lines, A2780 is derived from a serous subtype, MCAS from a mucinous subtype, ES2 from a clear cell subtype, and HO8910PM from an endometrioid tumor. These represent the four major histological ovarian carcinoma subtypes. And the subtype of SKOV3 is unclear. In contrast, the IC_50_ value of WG-391D for a normal ovarian epithelial cell line, HOSEPIC, was >33.33 μM (Figure 3A), indicating that WG-391D may be selectivity tolerant.

A total of eight different ovarian cancer cell lines (SKOV3, ES2, OVCAR8, OVTOKO, A2780, IGROV1, HO8910PM, and MCAS), including all four major histological subtypes of epithelial ovarian cancer, and four differing primary ovarian tumor cell lines (GFY004, CZ001, CZ006, and CZ008), were included in our experiments in order to confirm the high efficiency of WG-391D in terms of the inhibition of ovarian cancer cell proliferation. GFY004 and CZ006 are high grade serous carcinomas, CZ001 is a clear cell carcinoma, and CZ008 is a poorly differentiated carcinoma. As shown in Figure 3B, the IC_50_ values of WG-391D for inhibition in all of these cell lines were <10 μM. To analyze the correlations between CDC25B expression levels and the inhibitory rates of WG-391D, we correlated CDC25B mRNA expression levels and the IC_50_ of WG-391D. In all thirteen cell lines (SKOV3, ES2, OVCAR8, OVTOKO, A2780, IGROV1, HO8910PM, MCAS, HOSEPIC, GFY004, CZ001, CZ006, and CZ008), CDC25B expression levels trends to show a negative correlation with the IC_50_ of WG-391D (Figure 3C, left panel). These cells were then divided into two groups, the first group (OVCAR8, ES2, OVTOKO, A2780, HO8910PM, MCAS, GFY004, CZ001, and CZ006) featured low CDC25B expression levels and low WG-391D IC_50_. The second group (SKOV3, IGROV1, HOSEPIC, and CZ008) featured either high levels of CDC25B expression or high WG-391D IC_50_. Within each group, the CDC25B expression levels were statistically and negatively correlated to the WG-391D IC_50_ (Figure 3C, middle and right panel, respectively). This means that the inhibitory rate of WG-391D is positively correlated with CDC25B expression levels.

Cancer cell migration ability *in vitro* is positively correlated with cell invasion and metastasis *in vivo*. Here, we also evaluated the effect of WG-391D on the migration of ovarian cancer cells. The SKOV3 cell line exhibits strong migratory ability, and is one of the most studied cell models of epithelial ovarian carcinoma. As shown in Figure 3D, WG-391D significantly decreased SKOV3 cell migration at concentrations as low as 0.008 μM. The IC_50_ value of WG-391D for inhibition of SKOV3 cell migration was <0.2 μM (Figure 3D). To verify the efficiency of WG-391D in inhibiting ovarian cancer cell migration, three ovarian cancer cell lines (SKOV3, ES2, and OVCAR8), and three primary ovarian tumor cell lines (GFY004, GFY005, and CZ006), were included in the migratory assay. Cells were allowed to migrate for ~18 h in the presence of 1 μM WG-391D. As shown in Figure 3E, the IC_50_ values of WG-391D for the inhibition of cell migration were <1 μM in all six of these cell lines.

### WG-391D Induces Cell Cycle Arrest at the G2/M Phase and Induces Apoptosis in Ovarian Cancer Cells

**Figure 4:**
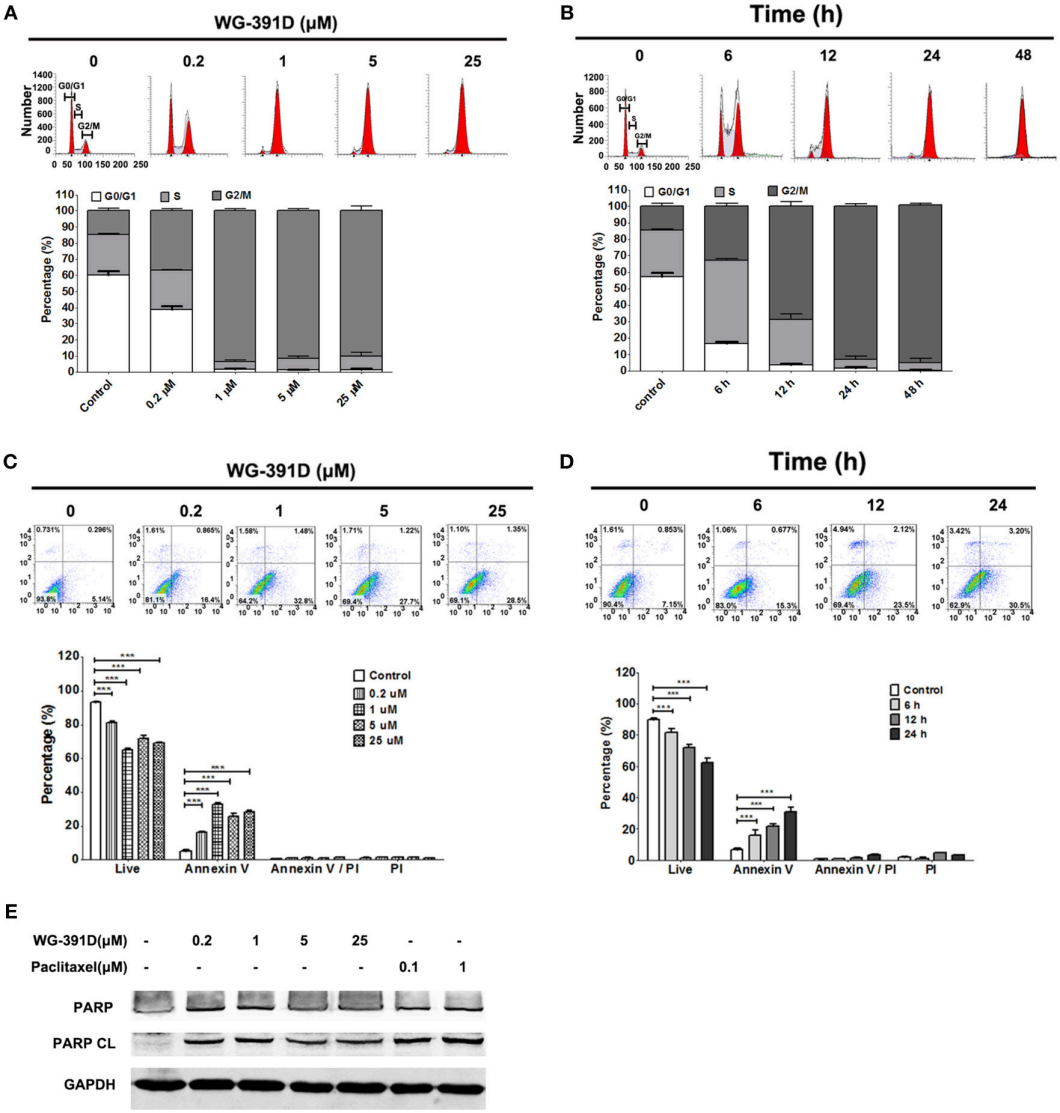
WG-391D induces ovarian cancer cell cycle arrest at G2/M phase and apoptosis. **(A**,**C)** SKOV3 cells were treated with the indicated concentrations of WG-391D for 24 h. **(B**,**D)** SKOV3 cells were treated with 5 μM WG-391D for different periods of time. **(A**,**B)** Cells were stained by PI and cell cycle phase distribution analyzed using ModiFit software. **(C**,**D)** Cells were stained using Annexin V and PI. Apoptotic cells were analyzed using Flowjo software. Upper panel, representative images; lower panel, statistical analysis of data. **(E)** Cells were treated with WG-391D, or paclitaxel, at the indicated concentrations for 24 h. PARP and cleaved PARP (PARP CL) were then detected by western blot assay. Total proteins (100 μg per sample) were analyzed. GAPDH served as a house-keeping control. ***p < 0.001.

The effects of WG-391D on ovarian cancer cell cycle progression and apoptosis were detected by FACS flow cytometry. Our data show that WG-391D inhibited SKOV3 cell cycle progression at the G2/M phase in a dose-dependent manner. WG-391D could strongly inhibit SKOV3 cell cycle progression at concentrations as low as 0.2 μM, when administered for 24 h (Figure 4A). Furthermore, WG-391D inhibited SKOV3 cell cycle progression in a time-dependent manner. WG-391D could induce cell cycle arrest in as short a time as 6 h at a concentration of 5 μM (Figure 4B). Moreover, WG-391D induced SKOV3 apoptosis in a dose- and time-dependent manner. WG-391D induced SKOV3 cell apoptosis at concentrations as low as 0.2 μM, when treated for 24 h (Figure 4C). Treatment with 5 μM WG-391D strongly induced cell apoptosis in as short a time as 6 h (Figure 4D). Cell apoptosis induced by WG-391D was also demonstrated by the induction of cleaved PARP in SKOV3 cells treated with WG-391D (Figure 4E). These data demonstrate that WG-391D causes SKOV3 cell cycle arrest and induces apoptosis in a dose- and time-dependent manner.

### WG-391D Inhibits Ovarian Tumorigenesis in Nude Mice

**Figure 5:**
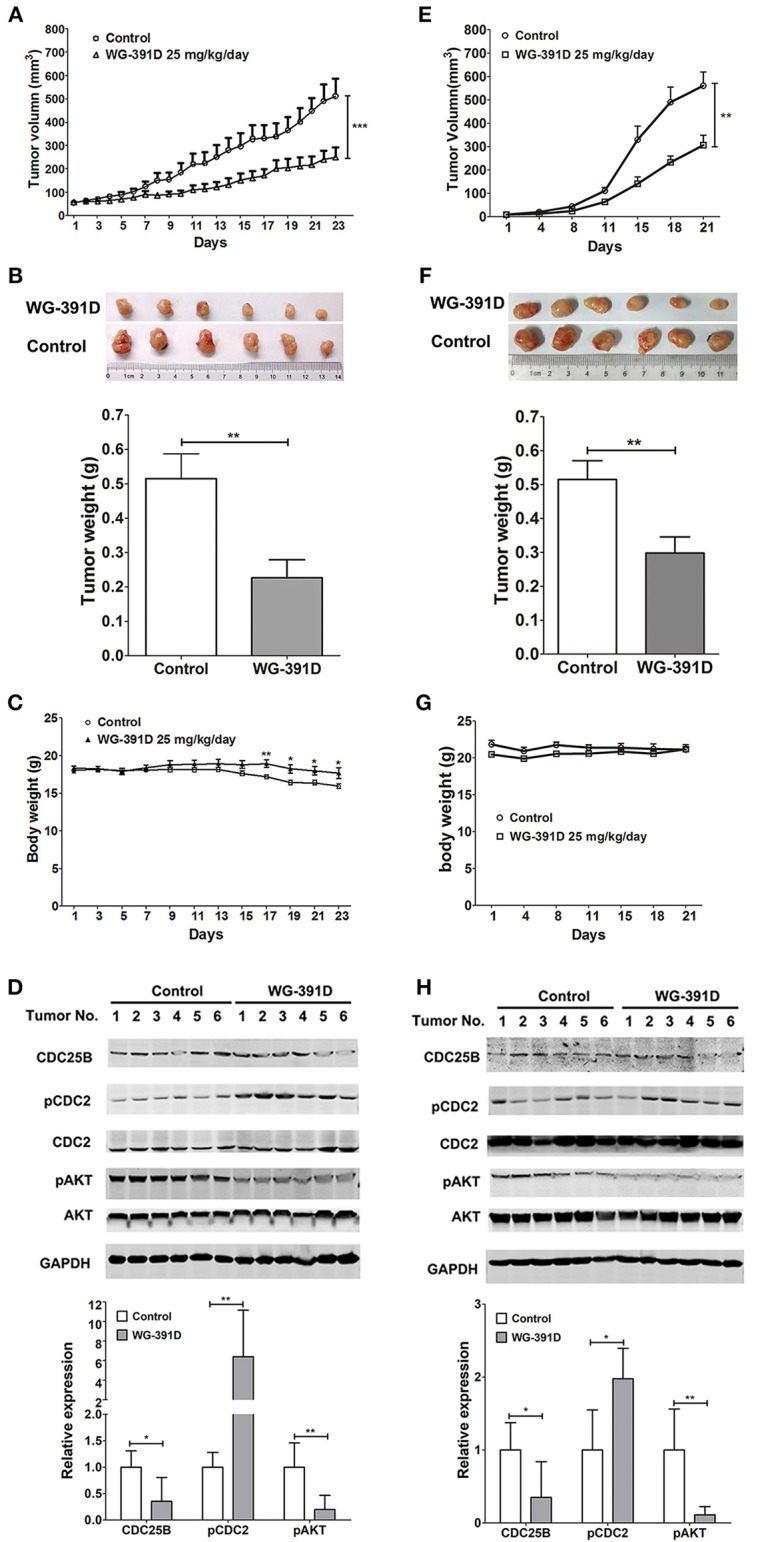
WG-391D strongly inhibits ovarian cancer tumorigenesis *in vivo*. BALB/c athymic nude mice were inoculated with 1 × 10^6^ SKOV3 cells **(A**–**D)**, or third generation PDX from patient GFY004 **(E**–**H)**, under the left front flank. Seven days after inoculation, mice were treated with 25 mg/kg/day WG-391D, or the same volume of DMSO as a negative control, for a further 3 weeks. Tumor volume **(A**,**E)** and mice body weights **(C** and **G)** were monitored on the indicated days. **(B**,**F)** Tumors were weighed after mouse were sacrificed. Upper panel, tumor images. Lower panel, statistical analysis of data. **(D**,**H)** Expression (CDC25B, CDC2, AKT, and GAPDH) and phosphorylation (pCDC2 and pAKT) of CDC25B, CDC2, and/or AKT in xenografts were detected by western blot (upper panel). Total proteins (100 μg per sample) were analyzed. GAPDH served as a house-keeping control. In the lower panel, band densities were measured by Image Pro Plus software. Differences of band densities between WG-391D treated groups and control groups were statistically analyzed. *p < 0.05, **p < 0.01, ***p < 0.001.

PDX models can better simulate the heterogeneity and genetic complexity of tumors *in vivo*. In creating our model, we were able to demonstrate similar morphology (e.g., glands, papillae, stromal cores, and desmoplastic stroma) of our PDX model across three generations (Figure S3). GFY004 is a high grade serous ovarian carcinoma and, as a PDX, grew very rapidly in nude mice and contained various types of cell with differing morphologies. Therefore, this PDX represented a good simulation of the heterogeneity and genetic complexity of high grade serous ovarian carcinoma. Five- to six-week-old female BALB/c athymic nude mice were inoculated under the left front flank with third generation PDX from GFY004, or with SKOV3 cells. Seven days later, mice were intraperitoneally administered with 25 mg/kg/day WG-391D for a further 3 weeks. Our results showed that tumor volume and tumor weight were inhibited by WG-391D in both SKOV3 cell xenografts (Figures 5A,B) and the PDX mouse models (Figures 5E,F). The inhibitory effect of WG-391D on ovarian cancer *in vivo* was also reflected by the inhibition of Ki67 expression in tumors from nude mice treated with WG-391D (Figure S2). WG-391D could protect tumor-bearing nude mice from body weight loss in the SKOV3 cell model (Figure 5C), while no significant change in body weight was observed in the PDX model (Figure 5G). Moreover, there were no obvious morphological changes in the lungs, liver and kidney of the experimental mice (data not shown).

In summary, WG-391D inhibited the expression of CDC25B, and inactivated both CDC2 and AKT in xenografts from nude mice inoculated with SKOV3 cells (Figure 5D), or a third generation PDX from patient GFY004 (Figure 5H).

## Discussion

Ovarian cancer is the fifth leading cause of cancer death among gynecological malignances, and has the highest mortality rate [~60% of all female tumors (1)]. More than two thirds of ovarian cancer patients have advanced disease at diagnosis, and almost all of them develop a recurrence after radical surgery and chemotherapy. Although a great deal of effort has been applied to the development of novel drugs and strategies for the treatment of ovarian cancer, the median 5-year OS rate has remained at a low level (30–40%) for the past two decades^1^. As ovarian cancer is a highly heterogeneous malignancy, a better understanding of its underlying mechanisms would greatly facilitate the search for superior rational and efficient target therapies.

Signaling molecules with key roles in ovarian cancer deterioration are of great significance as potential targets for the development of therapies for ovarian cancer. This study shows that CDC25B is strongly over-expressed in ovarian tumors compared with normal ovarian tissues and that CDC25B is universally strongly expressed in ovarian cancer and primary ovarian tumor cell lines. Consistent with our study, Broggini et al. also reported that CDC25B is universally expressed in ovarian cancer and associated with poor prognosis (35). Furthermore, CDC25B over-expression can activate AKT, and the co-inhibition of AKT and CDC25 has synergistic effects in the suppression of the growth of triple-negative breast cancer cells (34, 36). These studies highlight CDC25B as an attractive and rational target for cancer treatment (37).

Vitamin K analogs (23), NSC 663284 (24), naphthofurandione 3-benzoyl-naphtho[1,2-b]furan-4,5-dione (5169131) (25), adociaquinone B (26), IRC-083864, and BN82685 (27, 28) are the most potent CDC25B inhibitors reported to date; however, none of these compounds have entered clinical trials for ovarian cancer therapy. These inhibitors were all identified using *in vitro* chemical assays because of their high activity in inhibiting the catalytic dephosphorylation activity of CDC25B. Interestingly, a previous study reported that the compound CHEQ-2 not only inhibited CDC25A and CDC25B catalytic activity *in vitro*, but also caused down-regulation of CDC25A and CDC25B at doses higher than ~15 μM in human colon cancer (HT-29), breast cancer (MCF-7), and hepatocellular carcinoma (HepG2) cells (29). Importantly, this type of inhibitor could avoid the reversion of catalytic activity inhibition by high levels of CDC25 expression. When evaluating the ability of compounds from our laboratory's compound pool to down-regulate CDC25B by western blotting, we found that WG-391D could down-regulate CDC25B at doses as low as 0.2 μM, indicating that WG-391D was a powerful CDC25B inhibitor. Moreover, WG-391D was a CDC25B-specific inhibitor that did not affect the expression of CDC25A or CDC25C. All previously reported CDC25B inhibitors were not specific for CDC25B, and also exhibited inhibitory activity against CDC25A and/or CDC25C (37). Therefore, WG-391D could be valuable for the development of chemotherapeutics for cancer treatment. WG-391D is a highly efficient inhibitor of ovarian cancer cell proliferation and migration, with IC_50_ values <10 and 1 μM, respectively. The underlying mechanisms were associated with the down-regulation of CDC25B and the subsequent inactivation of CDC2 and AKT. Furthermore, we found that the inhibitory rates of WG-391D against proliferation correlated positively with CDC25B expression levels. WG-391D induces cell cycle arrest at the G2/M phase, and apoptosis. As more than 90% of ovarian cancers are high grade serous ovarian carcinomas, this study used a high grade serous ovarian carcinoma PDX model to evaluate the antitumor activity of WG-391D. In nude mice xenograft model experiments, we showed that WG-391D could significantly inhibit tumor growth in both SKOV3 cells and PDX mouse models. Furthermore, WG-391D could prevent the loss of body weight in mice. These data prove that WG-391D is an efficient inhibitor of ovarian cancer, with tolerable cytotoxicity.

**Figure 6:**
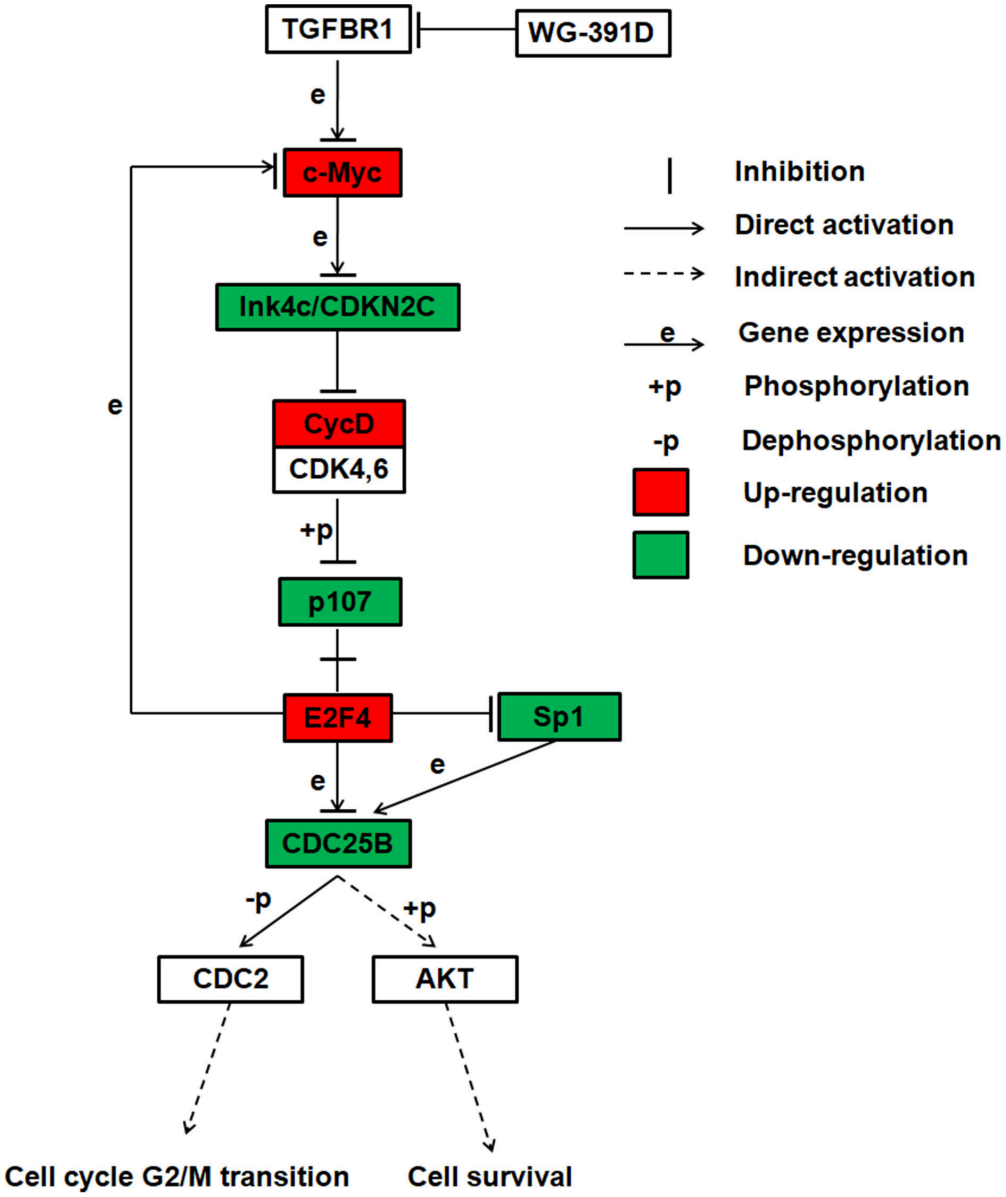
Summary of the proposed inhibitory mechanisms exhibited by WG-391D in ovarian cancer. This flowchart shows the most reliable signaling pathway inhibited by WG-391D.

WG-391D is a derivate of a compound known as YR-290 in our laboratory. Our group previously published a paper which demonstrated that YR-290 is an inhibitor of TGFβ Receptor 1 (TGFBR1) (38). In another paper, we also showed that the introduction of several simple tails on YR290 did not change its molecular target (30). WG-391D, as a derivative of YR290, featured the addition of only one amino alcohol tail substitution for a hydrogen atom, which means that compounds WG-391D and YR290 share the same scaffold. Consequently, the direct target of WG-391D should also be TGFBR1. Sp1 is an essential transcriptional promotion factor of CDC25B (39, 40), and E2F4 is a proven transcriptional repressor of CDC25B (41). The E2F4-p107 complex can directly inhibit Sp1 transcriptional activity by binding with Sp1 with p107 (42). According to our RNA sequencing data, Sp1 is highly down-regulated and E2F4 up-regulated by WG-391D. Also, a previous study showed that CDC25B could phosphorylate and activate AKT (34). Combining these previous publications and our present research data, we propose that WG-391D operates mainly through a signaling pathway involving TGFBR1-MYC-CDKN2C-CycD-p107-E2F4/Sp1-CDC25B-CDC2/AKT. The expression levels of genes associated with this pathway are shown in Table S1. This mechanistic model is also given as a flowchart in Figure 6. This study indicates that CDC25B down-regulators may be valuable for the clinical treatment of ovarian cancers.

## Conclusions

In conclusion, this study demonstrated that CDC25B was over-expressed in ovarian cancers. WG-391D efficiently inhibited ovarian cancer cell proliferation and migration, was associated with the induction of cell cycle arrest at G2/M phase and apoptosis, and inhibited the tumorigenesis of ovarian cancer in both SKOV3 cell and PDX BALB/c athymic nude mouse models. The anti-tumor activity of WG-391D involved down-regulation of CDC25B and subsequent inactivation of CDC2 and AKT. In conclusion, our data demonstrate that WG-391D has potential to be very valuable for the development of chemotherapeutic drugs for use in ovarian cancer therapy.

## Ethics Statement

Written informed consent was obtained from all individual participants. This study was conducted according to the Declaration of Helsinki and approved by the Ethical Committee of Fengxian District Central Hospital. All animal experiments were performed according to the Guidelines of the Laboratory Animal Ethical Board, and approved by the Animal Care and Use Committee of East China Normal University School of Life Sciences.

## Author Contributions

YX designed and performed the experiments and drafted the manuscript. YY, DG, WJ, YS, and PZ participated in the experiments. PJ, YL, CW, FG, CZ, and BW were involved in resource collection. RZ, BD, and YC supervised this study. All authors read and approved the final manuscript.

### Conflict of Interest Statement

The authors declare that the research was conducted in the absence of any commercial or financial relationships that could be construed as a potential conflict of interest. The reviewer SZ declared a shared affiliation, though no other collaboration, with several of the authors YS, PZ to the handling editor.
