# Supplementary material for: Inhibition of CDC25B With WG-391D Impedes the Tumorigenesis of Ovarian Cancer
Source: Front Oncol. 2019 Apr 8;9:236. doi: 10.3389/fonc.2019.00236 (PMC6463794; doi:10.3389/fonc.2019.00236)
Supplement: Supplementary file 1 [file Data_Sheet_1.doc]

Supplemental Table S1.

**Table S1. RNA sequencing data for the mRNA expression levels of several CDC25B-related genes.**

| **Gene ID** | **FPKM** | | | | | | **Fold change** | **p value** |
| --- | --- | --- | --- | --- | --- | --- | --- | --- |
|  | **Control** | | | **WG-391D** | | |  |  |
| CDC25B | 291.457 | 279.739 | 292.099 | 93.2645 | 86.1895 | 84.9751 | 0.3063 | 2.64E-16 |
| c-Myc | 24.8504 | 22.8457 | 19.2396 | 78.2864 | 78.2373 | 75.8464 | 3.4715 | 7.50E-30 |
| Ink4c/  CDKN2C | 33.6863 | 36.2492 | 34.9332 | 7.86764 | 6.03742 | 6.6207 | 0.1957 | 2.19E-26 |
| CycD/  CCND1 | 63.6659 | 55.9976 | 42.2199 | 118.069 | 148.152 | 148.947 | 2.5646 | 4.32E-19 |
| p107 | 2.38061 | 3.22042 | 2.77852 | 0.689443 | 1.24918 | 1.2483 | 0.3803 | 9.11E-10 |
| E2F4 | 32.4468 | 33.0327 | 33.0439 | 69.6822 | 63.6795 | 60.8355 | 1.9711 | 4.38E-12 |
| Sp1 | 17.6449 | 16.4639 | 11.6232 | 3.79176 | 6.14851 | 7.71311 | 0.3860 | 1.46E-06 |
| TGFBR1 | 2.01439 | 2.63121 | 2.2081 | 0.938954 | 1.77093 | 1.8469 | 0.6649 | 0.04372 |
| CDC25A | 5.34372 | 5.49483 | 4.24131 | 7.76393 | 7.28336 | 6.56416 | 1.4331 | 0.00014 |
| CDC25C | 10.3592 | 12.5395 | 11.6707 | 6.59934 | 8.59742 | 9.97044 | 0.7280 | 0.13861 |

Supplemental Figure S1.


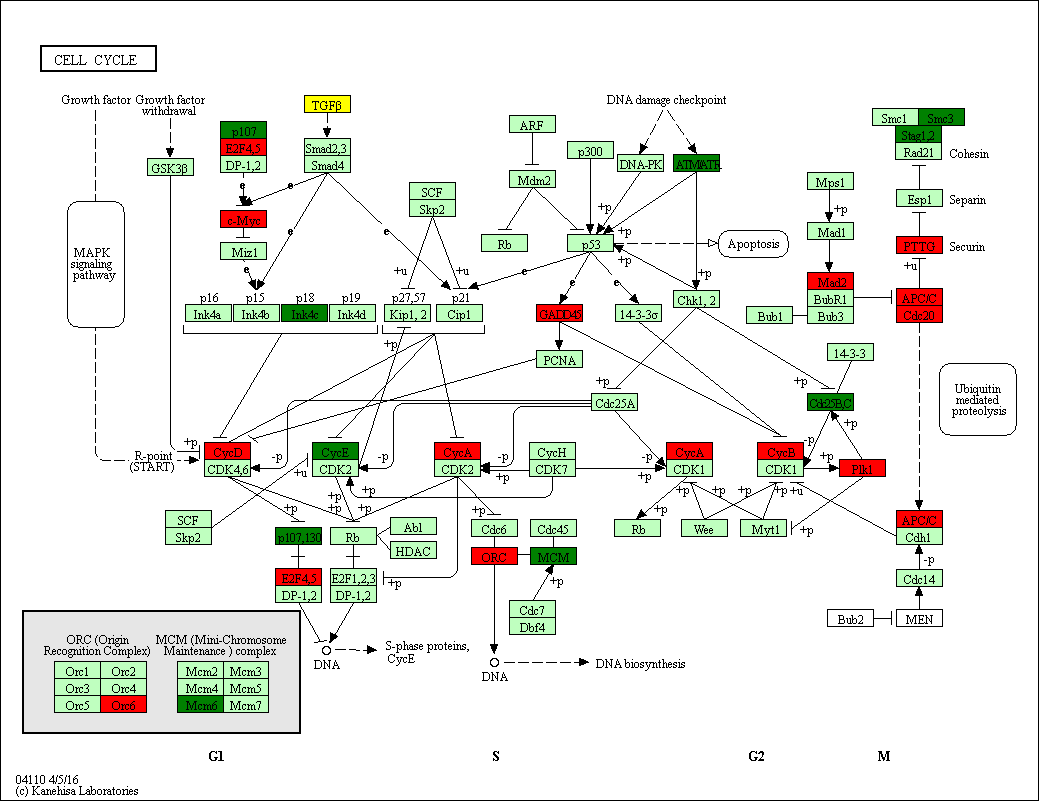


**Figure S1. KEGG pathway of the cell cycle.** Cell cycle pathway analysis by KEGG showed that, of all the genes associated with G2/M phase transition, only CDC25B was down-regulated by WG-391D.

Supplemental Figure S2.


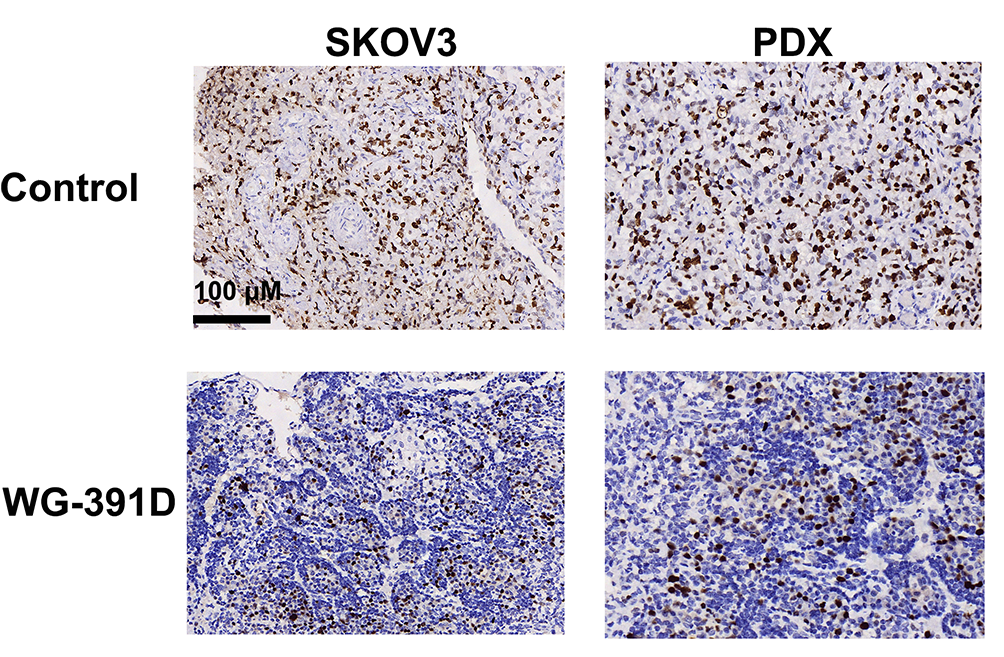


**Figure S2. WG-391D strongly inhibited Ki67 expression in both SKOV3 cell-derived tumors and PDX tumors *in vivo*.** Expression of Ki67 in tumors derived from nude mice, which had been treated with WG-391D, were detected by immunohistochemistry. Tumors derived from nude mice treated with the same volume of DMSO were used as negative controls.

Supplemental Figure S3.


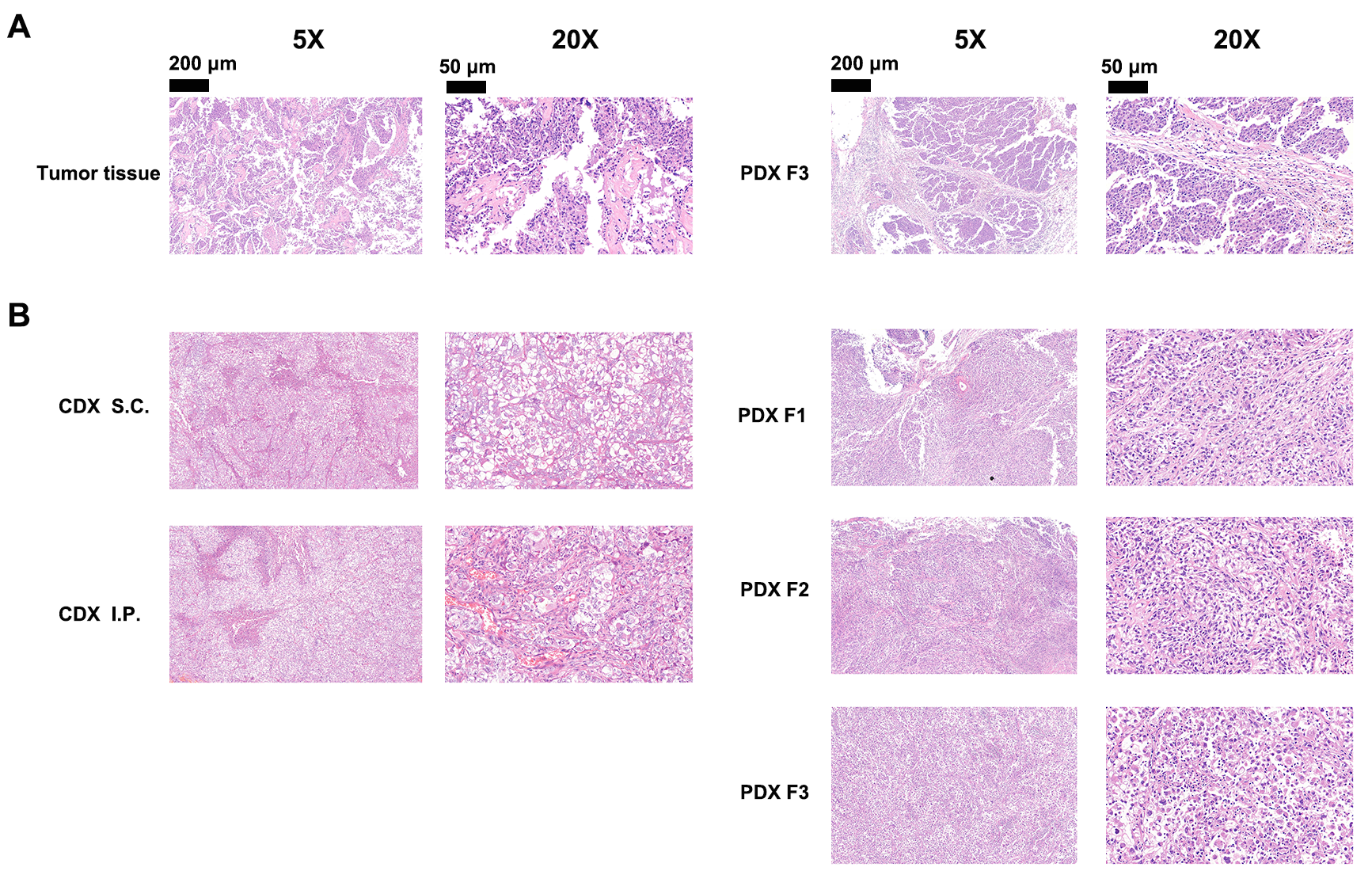


**Figure S3. Images demonstrating the morphology of PDX tumors was very similar to patient tumor tissues.** A: Hematoxylin and eosin (HE) staining of tumor tissues and PDX from the same patient showed similar morphology with respect to glands, papillae, stromal cores and desmoplastic stroma. B: HE staining of CDX or PDX from the GFY004 patient. For CDX creation, tumor cells from the GFY004 patients were isolated and cultured for approximately 20 generations *in vitro* and inoculated subcutaneously (S.C.) or intraperitoneally (I.P.) into BALB/c null nude mice at a concentration of 5 ×10^6^ cells. Six weeks later, mice were sacrificed and tumors were extracted. 5X: five-fold magnification. 20X: twenty-fold magnification. CDX: cell derived xenograft. PDX: patient derived xenograft. F1/F2/F3: First, second and third generation PDX, respectively.
